# Angiotensin-converting enzyme 2, the SARS-CoV-2 cellular receptor, is widely expressed in human myometrium and uterine leiomyoma

**DOI:** 10.1177/2284026520954068

**Published:** 2021-03

**Authors:** Alexon M Racilan, Wiviane A Assis, Maíra Casalechi, Ananda Spagnolo-Souza, Marcelo A Pascoal-Xavier, Ana C Simões-e-Silva, Helen L Del Puerto, Fernando M Reis

**Affiliations:** 1 Department of Obstetrics and Gynecology, Universidade Federal de Minas Gerais, Belo Horizonte, MG, Brazil; 2 Department of Pathologic Anatomy, Universidade Federal de Minas Gerais, Belo Horizonte, MG, Brazil; 3 Department of Pediatrics, Universidade Federal de Minas Gerais, Belo Horizonte, MG, Brazil; 4 Department of Pathology, Universidade Federal de Minas Gerais, Belo Horizonte, MG, Brazil

**Keywords:** ACE2, myometrium, uterine leiomyoma, fibroid, SARS-CoV-2, COVID-19

## Abstract

**Objective:**

Angiotensin-converting-enzyme 2 (ACE2), the cell surface receptor for severe acute respiratory syndrome coronavirus 2 (SARS-CoV-2), is found in a variety of reproductive tissues. The present study evaluated whether uterine fibroids and normal myometrium express ACE2 and, if so, at which tissue compartments.

**Methods:**

We included 13 premenopausal women (age range 33–50 years, median 40 years) with uterine fibroids undergoing elective hysterectomy or myomectomy. Samples of leiomyoma (*n* = 12) and normal myometrial tissue (*n* = 8) were analyzed by immunohistochemistry for protein localization or by real time PCR for mRNA detection.

**Results:**

In normal myometrium, ACE2 immunoreactivity was localized in smooth muscle fibers, arteriolar walls, and endothelial cells. In uterine leiomyoma, ACE2 staining was more intense in smooth muscle cells than in the extracellular matrix, and was also present in vascular endothelium. ACE2 mRNA was detected in myometrium as well as in fibroid samples.

**Conclusion:**

Human myometrium and uterine leiomyoma express ACE2 mRNA and have abundant distribution of ACE2 protein in their smooth muscle cells and microvasculature.

## Introduction

The coronavirus disease 2019 (COVID-19) starts by the entrance of SARS-CoV-2 into human cells, by binding to its cell surface receptor, the membrane bound form of a protein also known as type-2 Angiotensin Converting Enzyme (ACE2).^1^ We have previously demonstrated that ACE2 is present in a variety of reproductive tissues, such as testis,^2^ ovary,^3–5^ and endometrium, with predominance in the endometrial epithelium and during the secretory phase of menstrual cycle.^6^ Therefore, the human reproductive system is a potential target for SARS-CoV-2, but whether ACE2 is present in other uterine tissues like the myometrium remains unknown.^7^

If the myometrium and/or uterine leiomyomas express the protein ACE2, there is a plausible mechanism by which SARS-CoV-2 might infect these tissues, where it might trigger a local inflammatory response and might disrupt functional mechanisms of tissue repair.^8,9^ Inflammation in the myometrium may predispose to adenomyosis^10^ and also boost the growth of uterine fibroids, through paracrine actions of transforming growth factor β3 and proinflammatory cytokines released in the extracellular matrix.^11^ Therefore, the present study evaluated whether uterine fibroids and normal myometrium express ACE2 and, if so, at which tissue compartments.

## Methods

This cross-sectional study was approved by the Research Ethics Committee of Universidade Federal de Minas Gerais under protocol number CAAE 60375616.5.0000.5149, version 3. The participants signed an informed consent before being enrolled.

We included 13 premenopausal women (age range 33–50 years, median 40 years) with uterine fibroids undergoing elective hysterectomy or myomectomy for abnormal uterine bleeding between January and May 2019. Exclusion criteria were uterine atrophy, malformation or malignancy. Fibroid classification according to the FIGO criteria^12^ ranged from 2 to 4 and 54% of the participants were using progestins at the time of surgery. Samples of leiomyoma (*n* = 12) and normal myometrial (*n* = 8) tissue were obtained in the operating room. Half of the tissue fragments (six samples of fibroids and four of myometrium) were fixed in buffered formaldehyde and embedded in paraffin for subsequent use in immunohistochemistry, and the remaining samples were immediately immersed in RNA stabilization solution (RNAlater, ThermoFisher, São Paulo, Brazil).

Tissue sections of 5-μm thickness were mounted in silanized slides, deparaffinized in xylene, and rehydrated in serially diluted ethanol baths. Antigen retrieval was performed by microwave heating in EDTA buffer pH 8.0 for 5 min followed by cooling at room temperature. Using the Novolink™ non-biotin polymer detection system kit (Novocastra^®^, Newcastle Upon Tyne, UK), endogenous peroxidase activity was blocked with peroxidase block for 5 min, followed by 5 min incubation with protein block to reduce background staining. Next, sections were incubated with rabbit polyclonal antibody anti-human ACE2 (Abcam, Cambridge, UK, catalog number ab15348) 1:10 overnight at 4°C. Afterwards, post-primary block was added for 5 min to enhance penetration of the polymer reagent. Reactions were developed using 3,3′-diaminobenzidine and sections were counterstained with hematoxylin. Negative controls were processed with PBS instead of the primary antibody. High resolution images of the stained sections were acquired through a Pannoramic Digital Slide Scanner (3DHistech, Budapest, Hungary) and analyzed in full using CaseViewer 2.4 software, then representative areas were chosen to illustrate the findings.

Total RNA was isolated from samples using homogenization in Trizol reagent (Invitrogen, Carlsbad, CA, USA) and reverse transcription was performed on 1 μg of DNAse I treated RNA using SuperScript III reverse transcriptase kit (Invitrogen, Carlsbad, CA, USA). The cDNA was subsequently subjected to real time polymerase chain reaction using SYBR Green Master Mix kit (Life Technologies, Invitrogen, Carlsbad, CA, USA) as detailed elsewhere.^3^ The oligonucleotide primers used for human ACE2 (Accession number NM_021804.2) were 5′-GGATGGAGTACCGACT-3′ (forward) and 5′-TCCATTTACAGGCCCTTCTTCC-3′ (reverse). ACE2 gene expression was normalized by the reference gene *S26* (Accession number NM_001029.3, primer sequences 5′-CCGCCATCCGGCTAAATAGT-3′ and 5′-GGGTGGAAATGCGTTCCTAGT-3′) and the results were expressed as fold change (2^−ΔΔCT^).

## Results

**Figure 1 (a-d).**
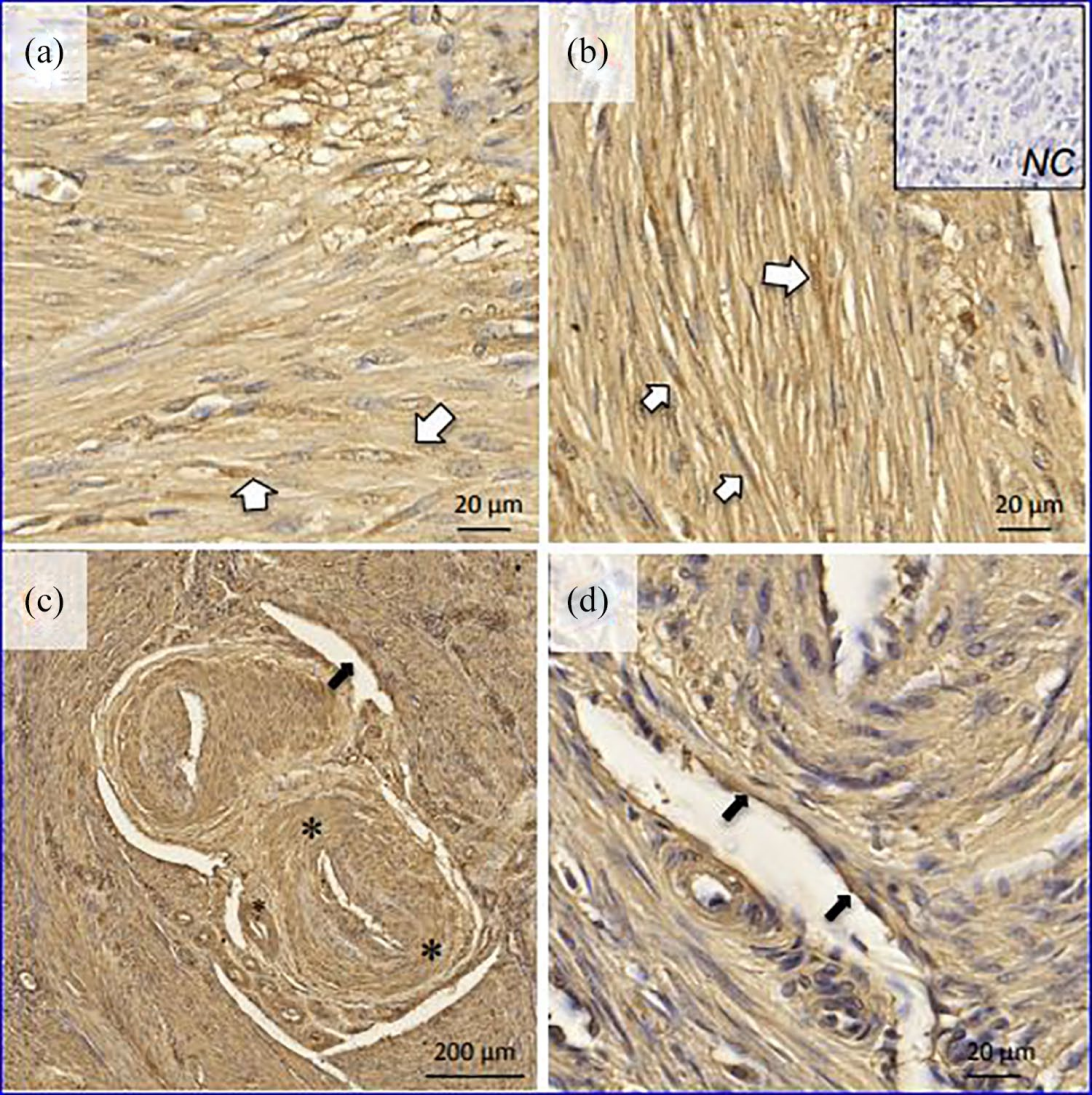
Localization of ACE2 in normal human myometrium by immunohistochemistry. White arrows: smooth muscle cells; black arrows: venous endothelial cells; asterisk: arteriolar wall. NC: negative control.

Figure 1 shows representative examples of ACE2 localization in normal myometrial tissue dissected out of hysterectomy specimens. There was diffuse immunostaining in smooth muscle fibers (Figure 1(a) and (b)), particularly around the nuclei, but also in the interstitial space. Of note, ACE2 immunostaining was evident in the circular layer of arteriolar smooth muscle (Figure 1(c)) as well as in the endothelium of small myometrial veins (Figure 1(d)).

**Figure 2 (a-d).**
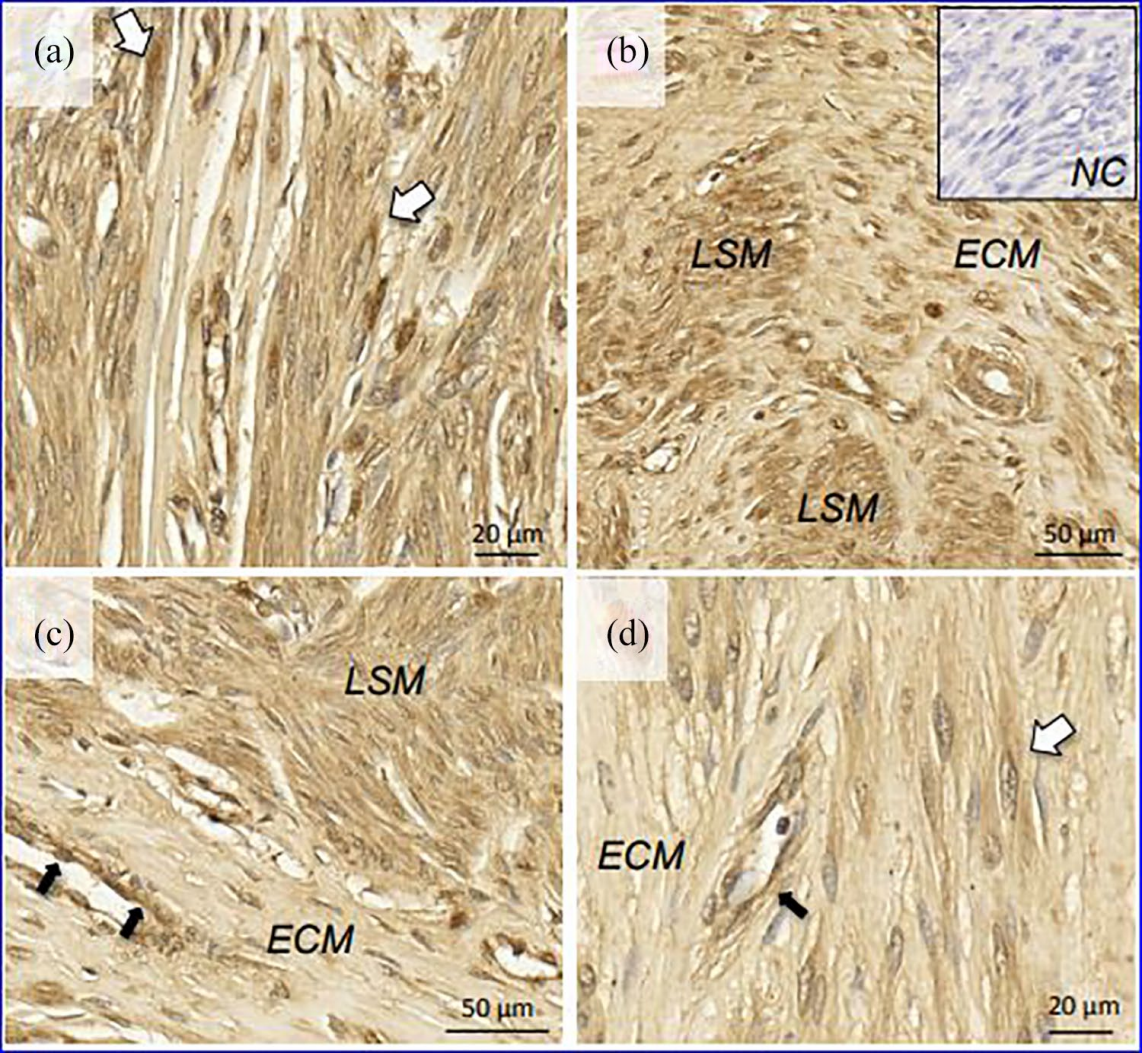
Localization of ACE2 in uterine leiomyoma by immunohistochemistry. White arrows: smooth muscle cells; black arrows: venous endothelial cells. LSM: leiomyoma smooth muscle cells; ECM: extracellular matrix – collagen fiber components between the cells; NC: negative control.

Uterine fibroids also had abundant expression of ACE2 (Figure 2). The protein was detected in leiomyoma cells characterized by elongated spindle shape nuclei, with indistinct cell borders and no evidence of mitotic activity, cellular and nuclear pleomorphisms (Figure 2(a)). In areas rich in extracellular matrix, ACE2 expression was much stronger in the leiomyoma smooth muscle fibers than in the surrounding collagen deposits (Figure 2(b) and (c)). Venous endothelia were also positive for ACE2 (Figure 2(c) and (d)).

**Figure 3.**
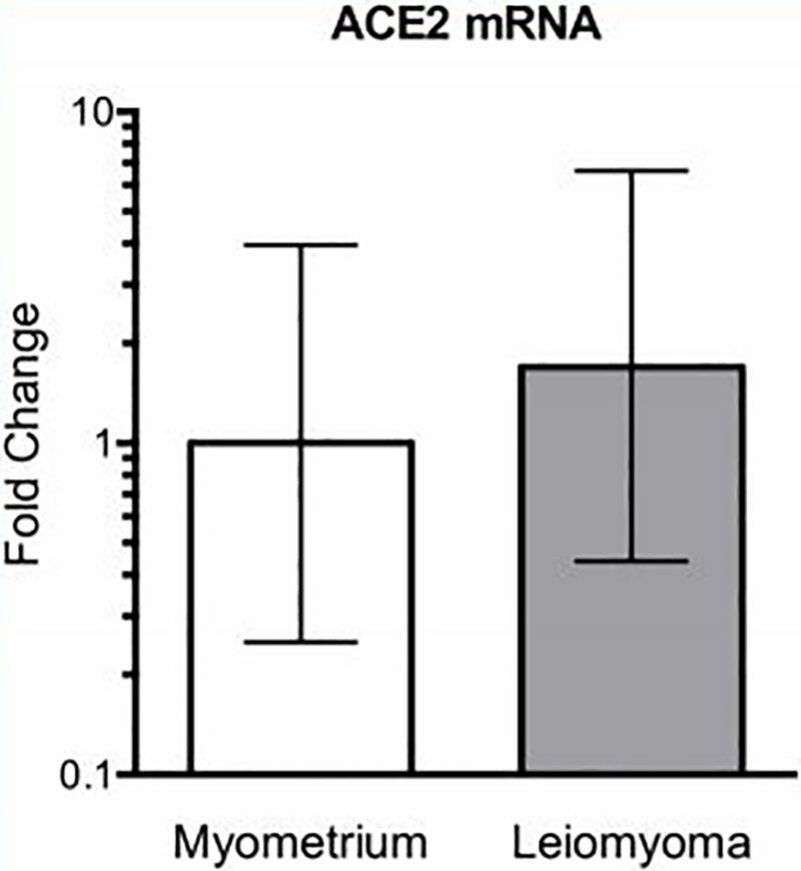
Relative expression of ACE2 mRNA in human myometrium (*n* = 4) and leiomyoma (*n* = 6).

ACE2 mRNA expression was quantified in myometrium and in uterine leiomyoma samples, although no mRNA quantitative difference between myometrium and leiomyoma was detected for ACE2 (Figure 3).

## Discussion

The present study investigated the mRNA expression and protein immunolocalization of ACE2 in uterine leiomyoma and normal myometrial tissue from premenopausal women. The results obtained clearly indicate the presence of ACE2 mRNA in both tissue types and show evidence of ACE2 localization in myometrium smooth muscle and blood vessels, as well as in leiomyoma cells.

While ACE2 had been detected in human endometrial cells,^6^ myometrial expression of ACE2 had been reported only in pregnant guinea-pig, particularly in the vascular smooth muscle of spiral, myometrial, and mesometrial arteries.^13^ In human, a previous study detected ACE2 mRNA in term gestation myometrial strips, but without protein assessment in this specific tissue.^14^ Thus, to the best of our knowledge, the present study is the first to show ACE2 expression and localization in non-pregnant human myometrium.

The potential implications of this finding reside, firstly, on the characteristic of ACE2 of being the main cell surface receptor for SARS-CoV-2, meaning that the presence of ACE2 is a precondition for a cell be susceptible to this type of coronavirus.^1^ However, another critical molecule to allow SARS-CoV-2 infection is the serine protease TMPRSS2, which is necessary for priming of the viral spike proteins.^15^ The presence of TMPRSS2 in human myometrium and uterine fibroids remains uncertain and therefore should be further assessed in conjunction with ACE2, as only cells co-expressing both proteins are potentially susceptible to SARS-CoV-2 entry.^15^ A second potential implication of the presence of ACE2 in human myometrium and uterine leiomyoma is the enzymatic activity after which ACE2 is named, that is, the cleavage of peptides from the renin-angiotensin system. ACE2 converts angiotensin II into angiotensin-(1–7), an heptapeptide that has anti-fibrotic properties in extra-reproductive tissues,^16^ a protective mechanism potentially relevant to stall the development of uterine fibroids^17^ and adenomyosis.^18^

This study has several limitations. We did not perform a quantitative comparison of ACE2 levels between normal myometrium and leiomyoma, but only a descriptive analysis of the protein distribution in the tissue compartments. The similar levels of ACE2 mRNA cannot be taken as conclusive evidence that myometrium and fibroids have the same levels of protein nor the same functional activity of ACE2 either as a SARS-CoV-2 receptor or as an angiotensin-(1–7) generating enzyme. The sample size did not confer statistical power to rule out a difference between myometrium and leiomyoma as regards ACE2 mRNA levels. In addition, the small sample size and the lack of quantitative protein measurements did not allow us to investigate whether ACE2 associates with clinical symptoms, fibroid topography, uterine volume, or preoperative medical treatments, which should be evaluated in the future. Nevertheless, as a proof of concept our results provide evidence that both normal myometrium and uterine fibroid possess ACE2 mRNA and protein and therefore fulfill one of the molecular conditions to be a SARS-CoV-2 target tissue.

In conclusion, human myometrium and uterine leiomyoma express ACE2 mRNA and have abundant distribution of ACE2 protein in their smooth muscle cells and microvasculature. It is still early to know whether women infected by SARS-CoV-2 will have any damage in their reproductive organs leading to a worse fertility prognosis^19,20^ or to severer fibroid symptoms. Nevertheless, prospective studies should assess these hypotheses since the virus is able to elicit intense inflammatory response in target tissues^9^ with still unknown sequelae.
